# The effectiveness of endoscopic ultrasonography findings to distinguish benign and malignant intraductal papillary mucinous neoplasm

**DOI:** 10.1007/s00464-022-09752-3

**Published:** 2023-03-07

**Authors:** Wu Dong, Ding Zhen, Wang Xiaoyan, Cheng Bin, Wang Ruifeng, Qin Shanyu, Li Zhuoran, Song Kai, Wu Wenming, Yang Aiming, Wu Xi

**Affiliations:** 1 Department of Gastroenterology, State Key Laboratory of Complex Severe and Rare Diseases, Peking Union Medical College Hospital and Chinese Academy of Medical Sciences, Beijing, 100730 China; 2 Division of Gastroenterology, Union Hospital, Tongji Medical College, Huazhong University of Science and Technology, Wuhan, 430022 China; 3 Department of Gastroenterology, Third Xiangya Hospital, Central South University, Changsha, 410013 China; 4 Department of Gastroenterology and Hepatology, Tongji Hospital, Tongji Medical College, Huazhong University of Science and Technology, Wuhan, 430030 China; 5 Department of Gastroenterology, The Fourth Hospital of Harbin Medical University, Harbin, 150001 China; 6 Department of Gastroenterology, The First Affiliated Hospital of Guangxi Medical University, Nanning, 530021 China; 7 Department of General Surgery, Peking Union Medical College Hospital and Chinese Academy of Medical Sciences, Beijing, 100730 China

**Keywords:** Pancreatic intraductal neoplasms, Endosonography, Pancreatic ducts

## Abstract

**Background and aims:**

Accurate evaluation of intraductal papillary mucinous neoplasm (IPMN) is necessary to inform clinical decision-making. But it is still difficult to distinguish benign and malignant IPMN preoperatively. This study aims to evaluate the utility of EUS to predict the pathology of IPMN.

**Methods:**

Patients with IPMN who underwent endoscopic ultrasound within 3 months before surgery were collected from six centers. Logistic regression model and random forest model were used to determine risk factors associated with malignant IPMN. In both models, 70% and 30% of patients were randomly assigned to the exploratory group and validation group, respectively. Sensitivity, specificity, and ROC were used in model assessment.

**Results:**

Of the 115 patients, 56 (48.7%) had low-grade dysplasia (LGD), 25 (21.7%) had high-grade dysplasia (HGD), and 34 (29.6%) had invasive cancer (IC). Smoking history (OR = 6.95, 95%CI: 1.98–24.44, *p* = 0.002), lymphadenopathy (OR = 7.91, 95%CI: 1.60–39.07, *p* = 0.011), MPD > 7 mm (OR = 4.75, 95%CI: 1.56–14.47, *p* = 0.006) and mural nodules > 5 mm (OR = 8.79, 95%CI: 2.40–32.24, *p* = 0.001) were independent risk factors predicting malignant IPMN according to the logistic regression model. The sensitivity, specificity, and AUC were 0.895, 0.571, and 0.795 in the validation group. In the random forest model, the sensitivity, specificity, and AUC were 0.722, 0.823, and 0.773, respectively. In patients with mural nodules, random forest model could reach a sensitivity of 0.905 and a specificity of 0.900.

**Conclusions:**

Using random forest model based on EUS data is effective to differentiate benign and malignant IPMN in this cohort, especially in patients with mural nodules.

Despite decades of research, pancreatic ductal adenocarcinoma (PDAC) remains the most aggressive solid malignancy with a 5-year survival of only 9% [1]. Intraductal papillary mucinous neoplasm (IPMN) is a well-documented precancerous lesion of PDAC. Probably due to the growing use of imaging examinations, patients with pancreatic cysts are increasingly detected, and IPMN accounted for 25%–38% of those cysts [2–5]. Histopathologically, IPMN was classified into low-grade dysplasia (LGD), high-grade dysplasia (HGD), and invasive carcinoma (IC) according to the Baltimore Consensus [6]. Different histological types of IPMN differ dramatically in prognosis and require different clinical management. It is reported that IPMN has an overall risk of developing PDAC of 2.8%, but high-risk IPMN (with dilated main pancreatic duct (MPD) or mural nodules) had a 5-year malignancy risk of 9.77% [7, 8]. Some clinical features and cysts features are associated with malignant IPMN, but it remains challenging to accurately categorize IPMN before surgery [9].

The revised Fukuoka Guideline recommended that IPMN with “high-risk stigmata” were indicated for surgical resection, while IPMN with “worrisome features” should undergo further evaluations, especially endoscopic ultrasonography (EUS), to determine the optimal therapeutic regimen [9]. Similarly, the European Guideline and American Gastroenterological Association (AGA) Guideline recommended surgery for those patients with positive cytology on endoscopic ultrasound-guided fine-needle aspiration (EUS-FNA), a solid component (mural nodule) or a dilated MPD [10, 11]. All three abovementioned guidelines emphasized the pivotal role of EUS in the evaluation of IPMN. The sensitivity and specificity of EUS ± FNA to diagnose malignant IPMN was 67.0%–75.6% and 70.0%–94.1%, respectively [12–14].

However, the preoperative detection of malignant IPMN and the surgical indication are still controversial. Most recommendations from current guidelines are based on low-quality evidence and expert’s consensus, and disagreement is common. For instance, MPD ≥ 10 mm is recommended in the 2012 International Guideline as an indication for surgery, but in the 2013 European Guideline the cutoff value of MPD width is 6 mm [9, 15]. The cancer risk in IPMN patients with an MPD diameter of 5-10 mm remained controversial until Hackert et al. reported that it did bear a significant risk of malignancy, and he suggested that surgical treatment should be considered when MPD > 5 mm [16]. Plus, a recent systematic review by Wu et al. suggested that MPD ≥ 5 mm in IPMN could be a sign of malignancy, and pancreatectomy is indicated for some patients [17]. Most existing studies in this field were single-center cohort without a validation group. Therefore, we conducted this multicenter study to evaluate the effectiveness of preoperative EUS in predicting malignant IPMN and determine the proper indication for surgical resection.

## Methods

### Patients population

In this retrospective multicenter study, we enrolled 115 patients from 6 medical centers who were diagnosed with IPMN by post-surgical pathology from January 2008 to October 2019. All the patients underwent EUS within 3 months before surgical treatment. Patients who had a pancreatic operation history were excluded in our study. Surgical pathology reports were reviewed by pathologists specialized in pancreatic diseases from each center who were blinded to clinical features and EUS findings. All the pathologists were uniformly trained and the pathological diagnosis was based on the Baltimore Consensus [6]. LGD was defined as benign IPMN, HGD and IC were defined as malignant IPMN in the present study. The Ethics Committee of Peking Union Medical College Hospital approved this study (ID: S-K937).

### Endoscopy techniques

Endoscopists with more than five years of experience performed EUS using the radial array echoendoscope technique (GF-UE260, 6/7.5 MHz, Olympus, Tokyo, Japan) that was connected to an endoscopic ultrasonic observation unit (EU-M2000, Olympus; EU-ME2 Premier plus, Olympus; Prosound F75, Aloka). De-aerated water was instilled to improve transmission of the US beam. During procedures, a radial endosonoscope was used to observe lesions, with endosono-staging starting from the default frequency of 7.5 MHz until satisfied imaging is obtained. A standard set of EUS images with pathological diagnosis were used to train and test endoscopists from each center. After passing the test, endoscopists from each center reviewed all EUS images and reports to extract valuable data and disagreements were solved by discussion with the senior authors (WX and YAM). All the endoscopists were blinded to the pathological classifications of the lesions. Description of pancreas morphology, cyst lesions, MPD, mural nodules as well as peripheral lymph nodes was recorded. The width of MPD was measured at the most dilated part of the main duct and size of cysts and mural nodules were defined as the largest diameter of the lesions.

### Statistical analysis

Continuous variables are exhibited as means and ranges and were compared using the Independent-Samples T-Test. Categorical variables were exhibited as frequency and proportion and compared using the chi-square or Fisher’s exact probability tests. To determine the optimal cutoff value of the width of MPD and the size of mural nodules, Youden index was calculated every variation of 0.5 mm. Univariate analysis and multivariate binary logistic regression models were used to explore the factors that may help distinguish the benign and malignant IPMN. We used simple random sampling provided by SPSS version 25.0 to choose 70% of our patients as an exploratory group to build up a predictive model for malignant IPMN. We draw the ROC curve and calculated the sensitivity and specificity. Then we used the remaining 30% data to validate the efficacy of our model. Data of the validation group was substituted into the logistic model to test our predictive model. To further improve the prediction significance of our model, we used random forest method in our analysis. Age, gender, smoking history, CA19-9, size of cysts, width of MPD, size of mural nodules, pancreatic atrophy, and lymphadenopathy were included in the model. Like the logistic regression model, we randomly chose 70% and 30% of the patients into the exploratory and validation group. Mean decrease accuracy and mean decrease Gini were calculated and dot chart was plotted to exhibit the importance rank of different variables. All reported P values were 2-sided with a value of 0.05. All the statistical analyses in our study were performed using SPSS version 25.0 and RStudio version 1.2.5033 for Windows.

## Results

### Patients’ characteristics

**Fig. 1:**
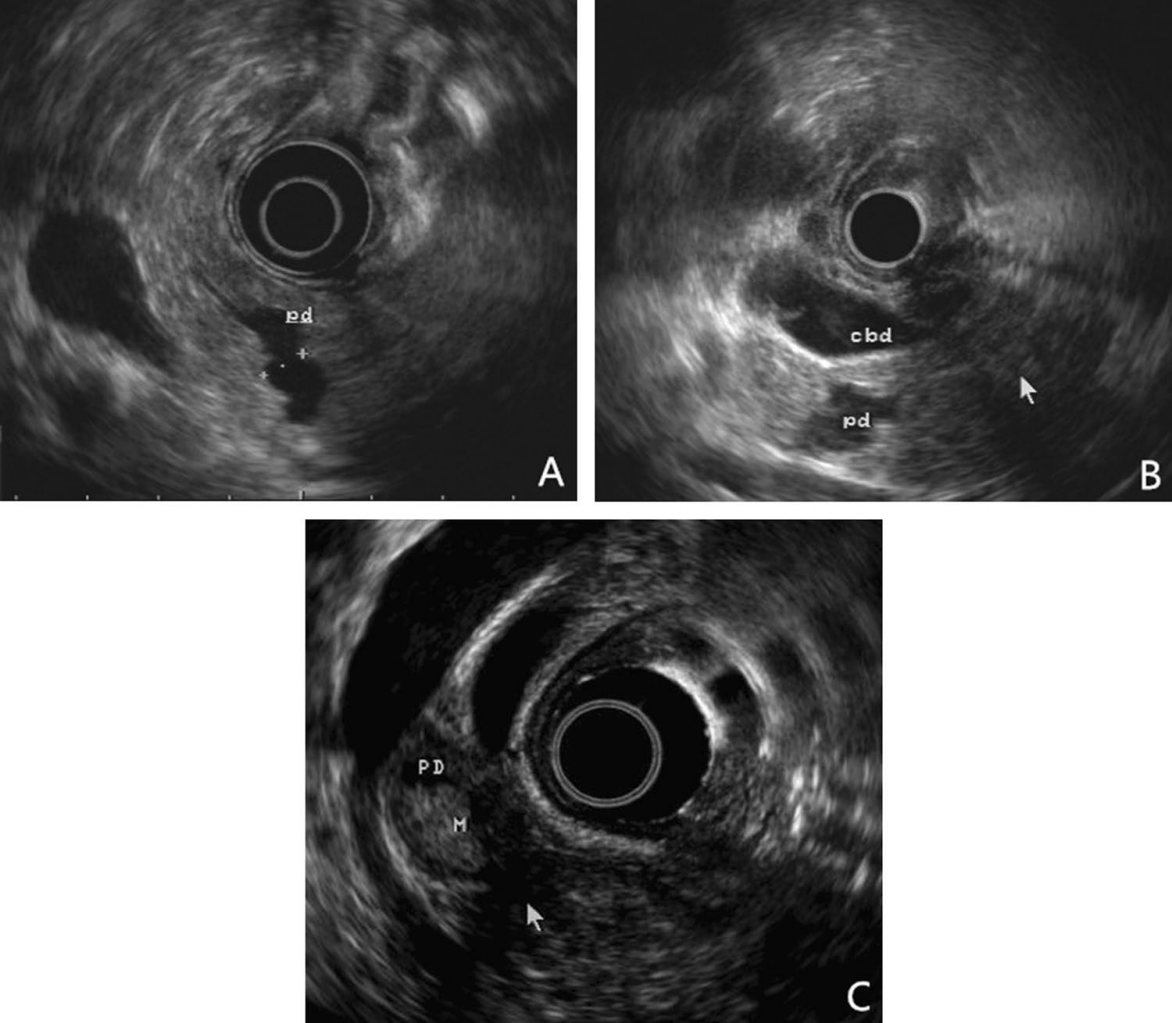
EUS images of IPMN with different pathology type. **A** Low-grade dysplasia. A cyst without mural nodules communicates with a non-dilated MPD. **B** High-grade dysplasia. A cystic communicates with dilated MPD without mural nodules. CBD is also dilated. **C** Invasive carcinoma. A mural nodule inside a dilated MPD. *EUS* endoscopic ultrasound, *IPMN* intraductal papillary mucinous neoplasm, *MPD* main pancreatic duct, *CBD* common bile duct

A total of 115 patients were enrolled in our study. Table 1 shows their demographic characteristics and clinical information. The 115 patients (including 78 males) had a mean age of 59.9 years (range 16–82 years). Abdominal pain was the most common chief complaint, which presented in 63 (54.8%) patients. Only a minority of patients had elevated pancreatic enzymes or tumor markers. Most lesions were MD-IPMN and located in the head of the pancreas. Therefore, the Whipple procedure was mostly used and other procedures included duodenum-preserving pancreatic head resection and subtotal resection of the pancreas. All patients survived surgical treatment but 36 (31.3%) patients developed major post-operative complications including pancreatic fistula (13, 11.3%), intra-abdominal infection (11, 9.6%), delayed gastric emptying (10, 8.7%), postpancreatectomy hemorrhage (8, 7.0%), biliary fistula (6, 5.2%), chyle leak (6, 5.2%), hospital-acquired pneumonia (5, 4.3%), acute kidney injury (4, 3.5%), and deep venous thrombosis (2, 1.7%). Patients with LGD, HGD, or IC were 56 (48.7%), 25 (21.7%), and 34 (29.6%), respectively. Figure 1 shows the typical EUS images of IPMN with different pathological types.

**Table 1:** Characteristics of IPMN patients (*N* = 115)

Characteristics of IPMN patients	Data(*N* = 115)
Demographic characteristics	
Age (years)	59.9 ± 11.2
Gender (male) (%)	78 (67.8)
Smoking history (%)	66 (57.4)
Alcohol history (%)	22 (19.1)
Family history of pancreatic cancer (%)	2 (2.6)
Clinical features	
Abdominal pain (%)	63 (54.8)
Medical examination (%)	27 (23.5)
Nausea/Vomiting (%)	4 (3.5)
Diarrhea (%)	5 (4.3)
Abdominal distension (%)	8 (7.0)
Others (%)	8 (7.0)
Laboratory examination	
Serum Amylase (> 125 U/L) (%)	12 (10.4)
Serum lipase (> 330 U/L) (%)	14 (12.2)
Total bilirubin (> 22.2 μmol/L) (%)	17 (14.8)
Direct bilirubin (> 8.6 μmol/L) (%)	22 (19.1)
CEA (> 5 ng/ml) (%)	23 (20.0)
CA19-9 (> 37 U/ml) (%)	21 (18.3)
Location	
Head (%)	72 (62.6)
Body/tail (%)	36 (31.3)
Diffuse (%)	7 (6.1)
IPMN type	
MD-IPMN (%)	74 (64.3)
MT-IPMN (%)	16 (13.9)
BD-IPMN (%)	25 (21.7)
Surgery	
Total pancreatectomy (%)	16 (13.9)
Whipple (%)	45 (39.1)
PPPD (%)	17 (14.8)
Distal pancreatectomy (%)	34 (29.6)
Others (%)	3 (2.6)

*CEA* carcinoembryonic antigen, *CA 19–9* carbohydrate antigen 19–9, *MD-IPMN* main duct IPMN, *MT-IPMN* mixed type IPMN, *BD-IPMN* branch duct IPMN, *PPPD* pylorus-preserving pancreaticoduodenectomy

### Predictors of malignant IPMN based on EUS findings

**Fig. 2:**
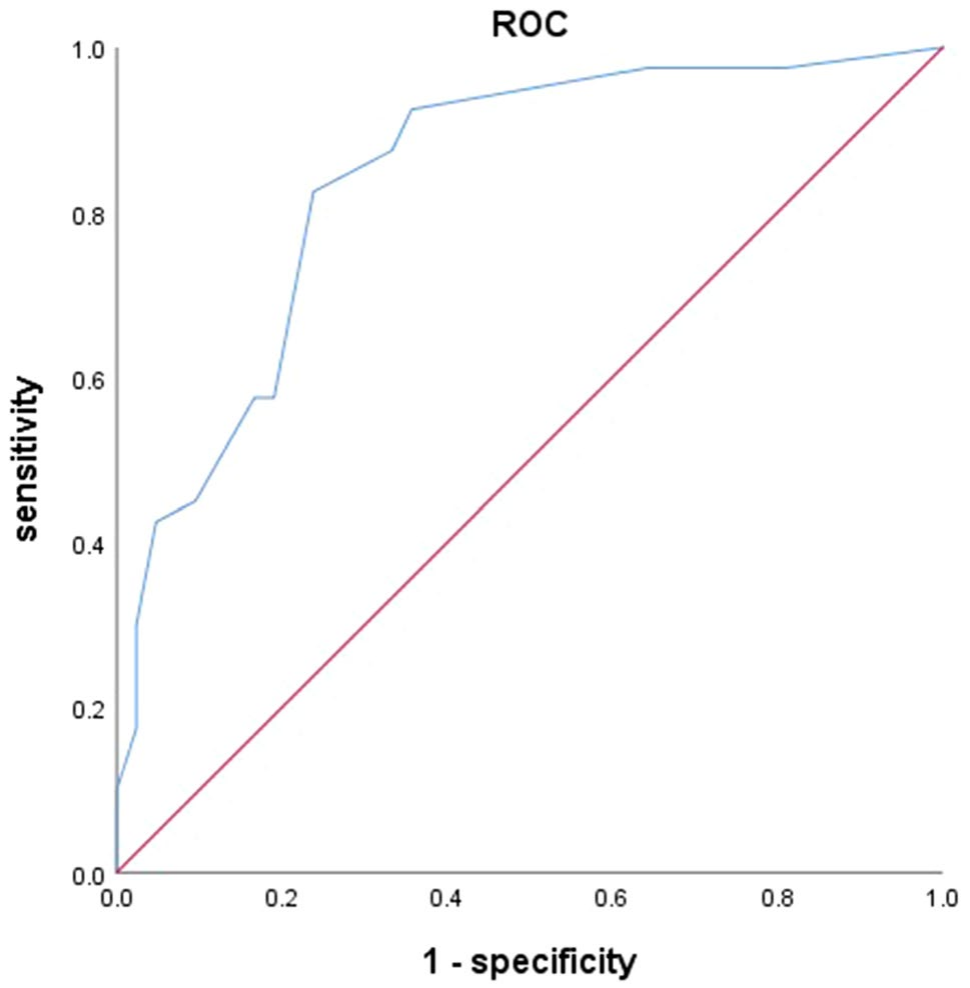
Receiver operating characteristic curve (ROC curve) of the exploratory group. Area under the curve (AUC) = 0.841

**Fig. 3:**
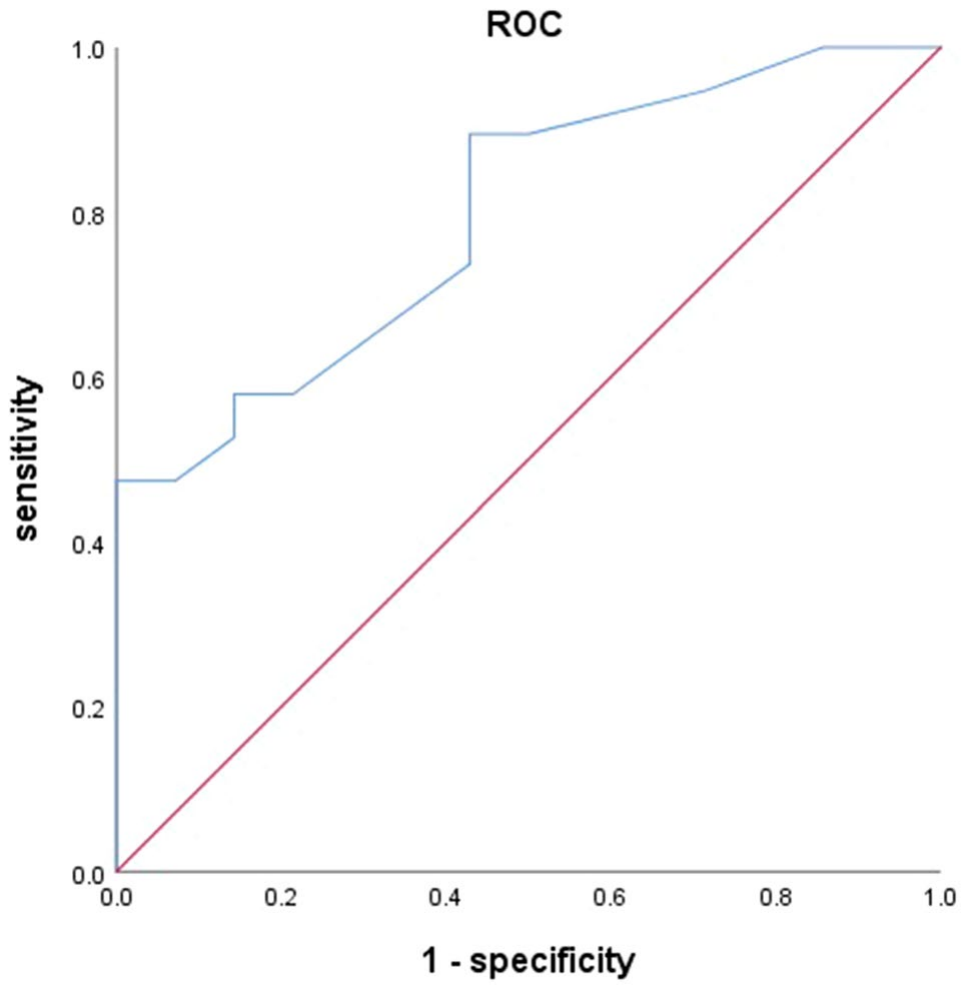
Receiver operating characteristic curve (ROC curve) of the validation group. Area under the curve (AUC) = 0.795

First, we found that MPD > 7 mm and mural nodules > 5 mm showed the best statistical differentiating performance with a Youden index of 0.320 and 0.307. Then, univariate analysis was performed to find the factors that might contribute to the diagnosis of malignant IPMN (Table 2). We found that proportions of male (*p* = 0.027), smoking history (p = 0.029), pancreatic atrophy (*p* = 0.027), lymphadenopathy (*p* = 0.026), MPD > 7 mm (*p* = 0.008), and mural nodules > 5 mm (*p* = 0.011) were significantly different between benign and malignant IPMN. Obstructive jaundice, elevated CA 19–9, and size of cysts did not show any significance. Gender, age, and other candidates screened by univariate analysis were involved in the binary logistic regression models (Table 3). Among them, we found that smoking history (OR = 6.95, 95%CI: 1.98–24.44, *p* = 0.002), lymphadenopathy (OR = 7.91, 95%CI: 1.60–39.07, *p* = 0.011), MPD > 7 mm (OR = 4.75, 95%CI: 1.56–14.47, *p* = 0.006) and mural nodules > 5 mm (OR = 8.79, 95%CI: 2.40–32.24, *p* = 0.001) were independently related with malignant IPMN. To better verify our results, we draw a ROC curve to evaluate the efficacy of EUS (Fig. 2). The sensitivity and specificity were 0.825 and 0.762, respectively, with an area under the curve (AUC) of 0.841. The remaining 30% data were also tested and we draw another ROC curve to compare the results of the exploratory and validation group (Fig. 3). We found that based on our model, the AUC of the validation group was 0.795. The sensitivity and specificity were 0.895 and 0.571, respectively.

**Table 2:** Univariate analysis of possible risk factors of malignant IPMN

Items (*N*=82)	Benign (*N*=42)	Malignant (*N*=40)	*P* value
Gender (male)	35 (83.3)	24 (60.0)	0.027
Age (years)	58.67±11.30	60.70±10.70	0.406
Smoking history	18 (42.9)	27 (67.5)	0.029
Alcohol history	8 (19.0)	8 (20.0)	1.000
Obstructive jaundice	3 (7.1)	2 (5.0)	1.000
Elevated CEA	9 (21.4)	7 (17.5)	0.783
Elevated CA 19-9	9 (21.4)	9 (22.5)	1000
Pancreatic atrophy	1 (2.4)	7 (17.5)	0.027
Thickened/enhanced cyst walls	6 (14.3)	6 (15.0)	1.000
Lymphadenopathy	4 (9.5)	12 (30.0)	0.026
Cysts>3cm	14 (33.3)	12 (30.0)	0.815
MPD>7mm	14 (33.3)	28 (70.0)	0.008
Mural nodules>5mm	9 (21.4)	20 (50.0)	0.011

*CEA*,carcinoembryonic antigen, *CA 19-9* carbohydrate antigen 19-9, *MPD*main pancreatic duct.

**Table 3:** Binary logistic regression on factors associated with malignant IPMN

	Odds ratio	95% CI	P value
Smoking history	6.95	1.98–24.39	0.002
Lymphadenopathy	7.91	1.60–39.07	0.011
MPD > 7 mm	4.75	1.56–14.47	0.006
Mural nodules > 5 mm	8.79	2.40–32.24	0.001

*MPD* main pancreatic duct

### Random forest model

**Fig. 4:**
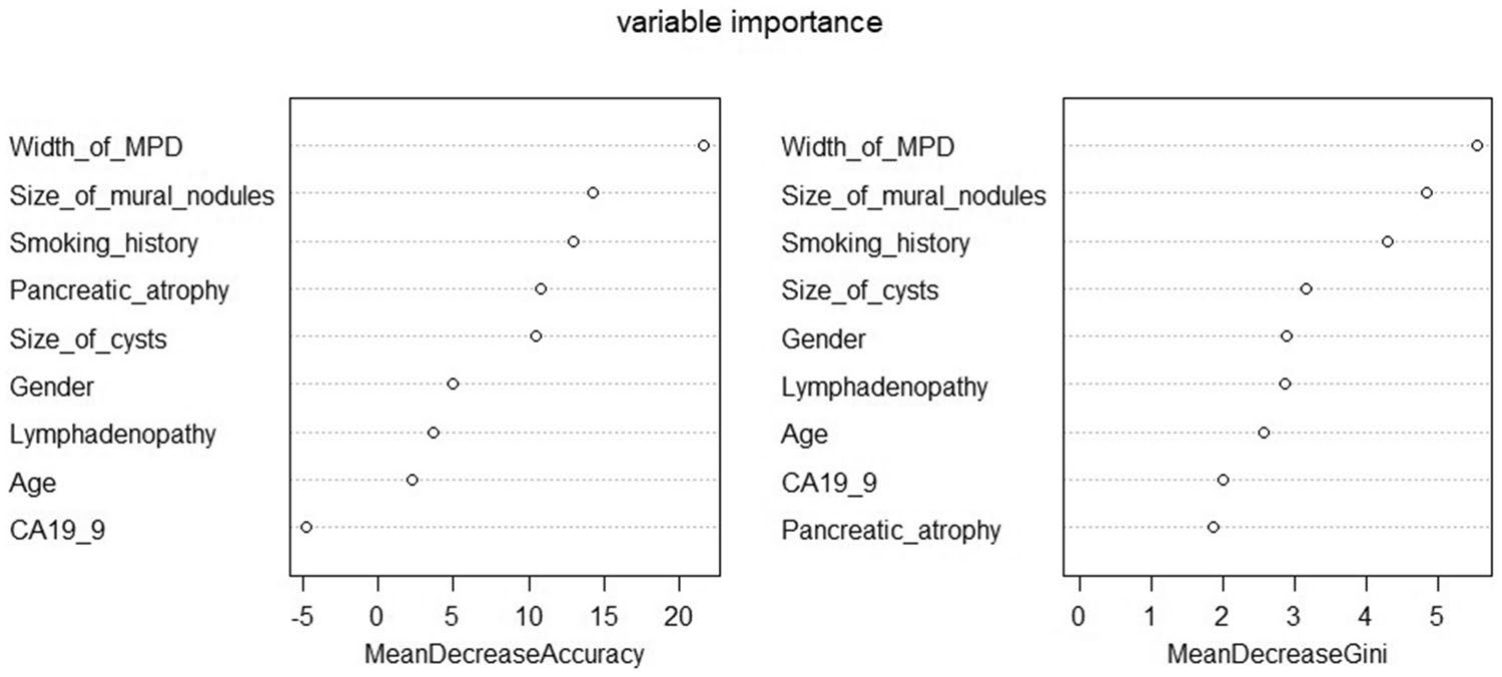
Mean decrease accuracy and mean decrease Gini of the random forest model

To further evaluate the predictive value of EUS, random forest model was used to distinguish benign and malignant IPMN. The sensitivity and specificity of the validation group were 0.722 and 0.823, respectively, with an AUC of 0.773. The importance rank of variables is shown in Fig. 4. Then, the same model was used in patients with mural nodules (N = 72) and acquired a sensitivity of 0.905 and a specificity of 0.900.

## Discussion

In the present multicenter study, we tried to identify the predicting factors associated with malignant IPMN. Several clinical and imaging findings including smoking history, lymphadenopathy, widened MPD and large mural nodules were identified as risk factors for malignant IPMN. Random forest model analysis showed that EUS could accurately diagnose malignant IPMN in patients with mural nodules.

According to our findings, MPD > 7 mm was the best cutoff value for distinguishing benign and malignant IPMN. European guidelines summarized previous studies concerning the relationship between the dilatation of MPD and the risk of malignant IPMN [10]. The cutoff value of the width of MPD ranged from 5 to 8 mm, and a recent study recommended a cutoff value of 6 mm [18]. Different cutoff values could be explained by different endoscope instruments and measurement error. Interestingly, we found that smoking history was one of the risk factors for HGD or IC. Nakagawa et al. demonstrated that in patients with IPMN, current smokers, but not former smokers had a greater chance of having PDAC concomitant with IPMN compared with non-smokers (OR = 4.9, 95% CI: 1.21–23.1, p = 0.03) [19]. Carr et al. found that smokers had a higher risk of early emergence of invasive IPMN, which indicated that cigarette smoking might be an accelerator in IPMN malignant progression [20]. In any case, cigarette quitting should be strongly recommended in patients with IPMN considering its carcinogenic effects. Large prospective epidemiological studies were still needed to verify the relationship between smoking and IPMN. Based on our results, mural nodules > 5 mm showed significance in differentiating benign and malignant IPMN. Some researchers suggested a mural nodule of 10 mm or larger was a predictor for malignancy and should undergo surgical resection [21, 22]. However, most studies supported the 5 mm cutoff value, which was accepted as one of the “high-risk stigmata” in the Fukuoka guidelines [9]. The appearance of lymphadenopathy was also considered a “worrisome feature” for IPMN, which was consistent with our result. Several previous reported “worrisome features” on EUS including thickened cyst walls, pancreatic atrophy and cysts > 3 cm were not significant in our study.

As most researchers have agreed, EUS seems to be a method with high sensitivity but low specificity [9, 23–26]. In the validation group of our logistic regression model, the sensitivity was high (0.895) but the specificity was low (0.571). Therefore, we tried another statistical method called random forest to optimize our model. Random forest is a machine learning method that can achieve maximum accuracy by systematically constructing multiple decision trees [27]. A recent study indicated that random forest showed better accuracy than logistic regression in most binary classification settings, especially for prediction [28]. In our study, random forest model achieved a modest sensitivity (0.722) and higher specificity (0.823). As known, mural nodules could be found in about 90% HGD and nearly all IC in the resected lesions [9]. However, the detection of mural nodules on images was not robust enough to distinguish benign and malignant IPMN, partially because small mural nodules were easily confused with mucus in the cyst [29]. So, we assumed that the diagnostic efficacy might increase if we combine the high sensitivity of mural nodules and the high specificity of our model. As expected, the sensitivity and specificity were both reached 0.900 in patients with mural nodules. From a clinical perspective, our random forest model can help to predict the pathology of IPMN with mural nodules preoperatively.

Several new methods have been applied to further evaluate IPMN. EUS-FNA might be a reasonable choice given its high specificity [30, 31]. One meta-analysis reported the sensitivity and specificity of EUS-FNA were 0.648(95%CI: 0.44–0.82) and 0.906 (95%CI: 0.81–0.96) [32]. But it was technically demanding to obtain enough tissue. Complications associated with EUS-FNA should also be a concern for clinicians [33]. Contrast-enhanced endoscopic ultrasonography was more sensitive in detecting mural nodules and could distinguish tissues from mucus, but it wasn’t widely used in some centers [34, 35]. A series of other methods such as pancreatoscopy, cyst juice analysis, and detection of k-ras mutation have been recommended in the updated European Guideline [10]. Machine learning and artificial intelligence could also be used in the diagnosis of IPMN. Recently, Kuwahara et al. reported that artificial intelligence via deep learning algorithms reached an accuracy of 0.940 in predicting malignant IPMN in a small group of patients, much higher than human diagnosis [36]. We believe that a more individualized and comprehensive evaluation of IPMN will become the mainstream for preoperative evaluation of IPMN.

Our study has some limitations. First, this is a retrospective study and certain selection bias is inevitable. But we collected data from multiple centers to increase the generalizability of our results. Second, EUS was performed by different endoscopists in different centers, and they were not blinded to the previous examination results. However, all endoscopists have qualified skill, and EUS images were reviewed in each center to minimize the bias. Third, our sample size was relatively limited, especially in the validation group. The predictive model needed to be tested in a separate prospective cohort in future studies. Fourth, branch duct IPMN (BD-IPMN), bearing a much different natural history than main duct IPMN (MD-IPMN) or mixed type IPMN (MT-IPMN), has a much lower risk to develop invasive cancer than the other two types. However, although including BD-IPMN may add unnecessary heterogeneity to the study, a very recent multicenter study enrolling 837 patients with BD-IPMN demonstrated that 168 patients (20%) developed worrisome features/high-risk stigmata, out of which 18 patients (11%) were proved to have high-grade dysplasia or invasive cancer from surgical resection samples [37]. This study indicates that the risk of progression in BD-IPMN is low but not negligible. Besides, the European Guideline and the AGA Guideline differ with regard to the optimal surveillance strategy of BD-IPMN, hinting lack of robust evidence in this field. Therefore, at this juncture we believe that it makes sense to include BD-IPMN patients in this study and will consider restricting the crowds to MD-IPMN or MT-IPMN to produce more homogenous evidence in our future studies. Last but not least, contrast-enhanced EUS has improved efficacy to observe IPMN, particularly mural nodules, and we will evaluate it in future studies.

In conclusion, EUS helped to distinguish benign and malignant IPMN. Random forest predictive model showed high accuracy in IPMN with mural nodules. Novel techniques and statistical method will help clinicians to manage patients with IPMN.
